# Sextus chest radiograph severity score correlates to clinical outcomes in patients with COVID-19: A cross-sectional study

**DOI:** 10.1097/MD.0000000000027663

**Published:** 2021-11-12

**Authors:** Justin Sun, Daohai Yu, Kevin Yoo, Robert Choi, Xiaoning Lu, Taylor Standiford, Gary Cohen, Nathaniel Marchetti, Omar Agosto, Maruti Kumaran, Hillel Maresky

**Affiliations:** a Temple University Lewis Katz School of Medicine, Philadelphia, PA; b Department of Radiology, Temple University Hospital Network, Philadelphia, PA; c Department of Pulmonology, Temple University Hospital Network, Philadelphia, PA.

**Keywords:** coronavirus disease-2019 imaging, CXR-CT correlation, reproducibility, serial chest radiography, Sextus score

## Abstract

The value of chest radiography (CXR) in detection and as an outcome predictor in the management of patients with coronavirus disease-2019 (COVID-19) has not yet been fully understood.

To validate a standardized CXR scoring system and assess its prognostic value in hospitalized patients found to have COVID-19 by imaging criteria and to compare it to computed tomography (CT).

In this cross-sectional chart review study, patients aged 18-years or older who underwent chest CT at a single institution with an imaging-based diagnosis of COVID-19 between March 15, 2020 to April 15, 2020 were included. Each patient's CXR and coronal CT were analyzed for opacities in a 6-zonal assessment method and aggregated into a “Sextus score.” Inter-reader variability and correlation between CXR and coronal CT images were investigated to validate this scoring system. Univariable and multiple logistic regression techniques were used to investigate relationships between CXR scores and clinical parameters in relation to patient outcomes.

One hundred twenty-four patients (median [interquartile range] age 58.5 [47.5–69.0] years, 72 [58%] men, 58 [47%] Blacks, and 35 [28%] Hispanics) were included. The CXR Sextus score (range: 0–6) was reliable (inter-rater kappa = 0.76; 95% confidence interval [CI]: 0.69–0.83) and correlated strongly with the CT Sextus score (Spearman correlation coefficient = 0.75, *P* < .0001). Incremental increases of CXR Sextus scores of 2 points were found to be an independent predictor of intubation (adjusted odds ratio [95% CI]: 4.49 [1.98, 10.20], *P* = .0003) and prolonged hospitalization (≥10 days) (adjusted odds ratio [95% CI]: 4.06 [1.98, 8.32], *P* = .0001).

The CXR Sextus score was found to be reproducible and CXR-CT severity scores were closely correlated. Increasing Sextus scores were associated with increased risks for intubation and prolonged hospitalization for patients with COVID-19 in a predominantly Black population. The CXR Sextus score may provide insight into identifying and monitoring high-risk patients with COVID-19.

## Abstract 2

Supplemental Digital Content is available in the text

## 1 Introduction

First reported from Wuhan, China in December 2019, a novel strain of coronavirus (SARS-Cov-2) was isolated from patients experiencing a febrile respiratory tract illness now known as coronavirus disease-2019 (COVID-19).^[1]^ As of August 2020, the number of confirmed COVID-19 cases has reached more than 21 million cases globally.^[2]^ Despite the global declaration of pandemic, enforcement of strict quarantine rules, and travel restrictions, COVID-19 continues to spread rapidly and additional surges appear imminent.^[3]^

With the rapid influx of patients experiencing respiratory tract symptoms and finite medical resources, there have been limitations in diagnostic capabilities especially in endemic areas (i.e., “hot spots”) during heightened COVID-19 spikes.^[4]^ Chest computed tomography (CT), with its sensitivity of up to 96%,^[5,6]^ is currently considered one of the most reliable methods for the triage of possible COVID-19 patients.^[7–10]^ The American College of Radiology and British Society of Thoracic Imaging have recognized the value of CT imaging and chest radiography (CXR) when used appropriately while cautioning against the use of CT in large-scale screening or first-line testing to diagnose COVID-19.^[11,12]^

CXR, which has hitherto taken a back seat to CT scanning in COVID-19 positive or suspected patients, is still one of the most commonly performed radiological investigations due to its versatility and broad indications. Despite the reported lower sensitivity (57%–69%) of CXR for detecting COVID-19,^[13–15]^ frequent CXRs may have a promising role both in triaging patients and monitoring evolving pulmonary abnormalities in critically ill patients. Recent reports have identified correlations between disease severity and CXR findings and underscored the value of early imaging in COVID-19 patients.^[14,16,17]^ Although a recent Cochrane review of 2 trials suggested an equivocal role of routine CXR in patients with lower respiratory tract infections, the significance of CXR in assessing disease severity and prognosis in patients with COVID-19 warrants additional investigation.^[18,19]^

Extracting additional objective information from CXRs to better monitor and identify high risk patients could improve patient management especially during high-volume situations. The purpose of our study was to assess the prognostic value of CXR using a standardized scoring system in hospitalized patients found to have COVID-19 by imaging criteria, to test its reproducibility, and to compare this score to CT.

## 2 Materials & methods

This retrospective cross-sectional study was approved by the Institutional Review Board of Temple University Health Network. The requirement for informed consent was waived. Clinical and imaging data were aggregated from the electronic medical record and used to validate a 2-step 6-zonal assessment scoring system on CXRs and investigate correlating clinical outcomes.

### 2.1 Inclusion and exclusion criteria

**Figure 1:**
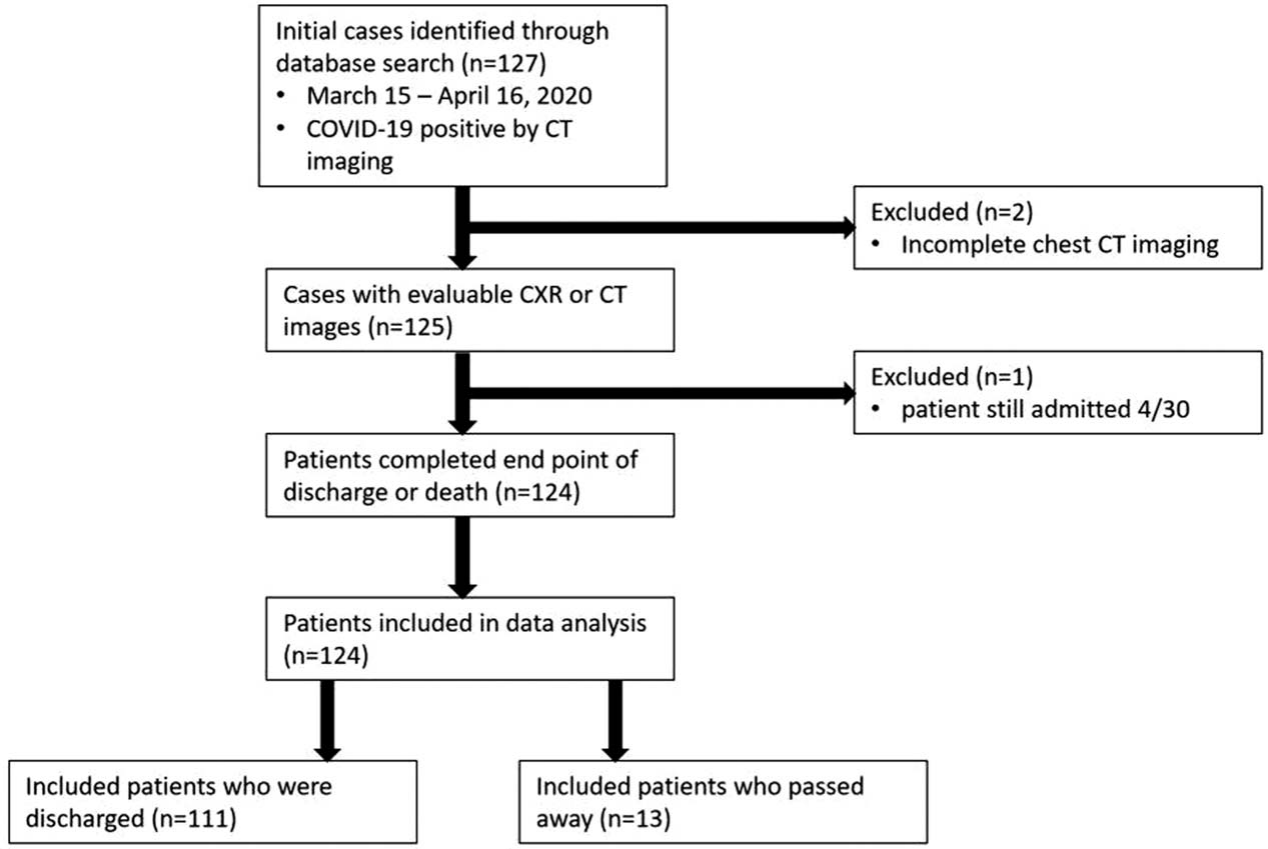
Flow diagram of cross sectional cohort study illustrating inclusion and exclusion criteria.

All patients with the diagnosis of COVID-19 and who were admitted to TUHN a large urban multicenter health system from March 15 to April 16, 2020 were included in our study. Inclusion criteria were age 18 years or older, positive COVID-19 imaging findings and acquisition of both chest CT and CXR. Patients with unevaluable images or who did not meet the primary endpoints of discharge or death by April 30, 2020 were excluded as study analyses began May 1^st^. After exclusions, 124 patients were included for analysis (Fig. 1). Patients with multiple admissions during this period had their clinical and imaging data combined from each admission.

### 2.2 Clinical data collection

Data extracted from the patients’ electronic medical record included age, sex, race, body mass index (BMI), smoking status (never-smoker, current smoker, former smoker, unknown), comorbidities (hypertension, diabetes mellitus type II [T2DM], chronic obstructive pulmonary disease, cardiovascular disease, malignancy, cerebrovascular disease, chronic kidney disease, asthma, hyperlipidemia, chronic liver disease, history of lung transplant), level of care (isolation ward, intensive care unit [ICU]), length of ICU stay, length of hospitalization, intubation status, tracheostomy status, duration on ventilator, inpatient endpoint (discharge, death), leukocyte counts, C-reactive protein levels, lactate dehydrogenase levels, and reverse transcriptase-polymerase chain reaction (RT-PCR) testing results. Patients who had a positive RT-PCR test at any time during their admission were considered RT-PCR positive for COVID-19. Prolonged length of hospitalization was defined as ≥10 days.

### 2.3 Imaging data collection

All patients either received digital portable anteriorposterior CXRs or digital posterioranterior/lateral CXRs and at least 1 chest CT. One CXR per day per patient (first CXR of the day), a chest CT coronal section centered around the carina for each CT per patient, and their corresponding CT trinary category impressions (category 1 = consistent with viral/atypical pneumonia, category 2 = indeterminate, category 3 = consistent with other diagnosis) were obtained from the picture archive and communications system and imported into a database for analysis.

### 2.4 Imaging analysis

**Figure 2:**
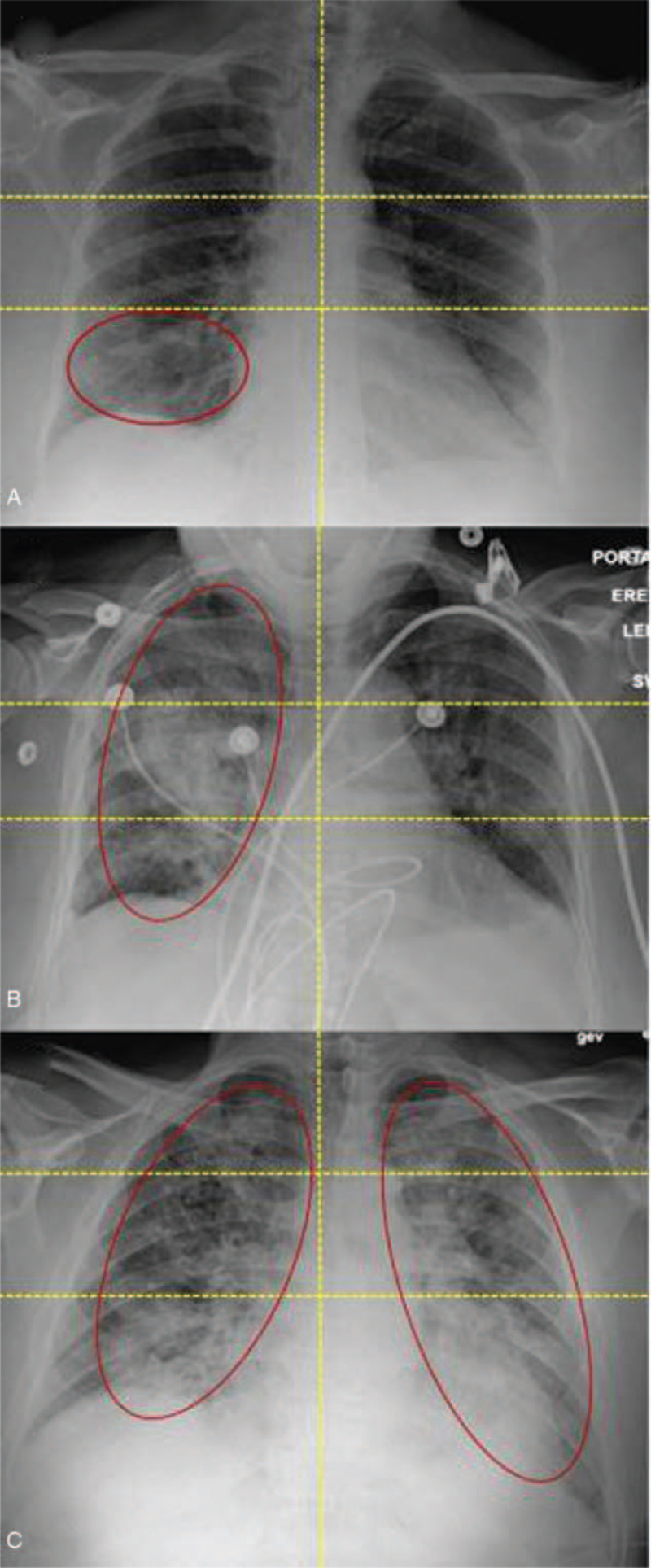
Examples of chest radiography (CXR) Sextus scores. (A) CXR of a 78-year old woman with a history of morbid obesity (BMI = 43), hypertension, and hyperlipidemia who presented with shortness of breath. Portable CXR shows left lower lobe opacity with a total Sextus score = 1 with opacities in the RLL (right lower lobe). (B) CXR of a 67-year old woman with a history of hypertension, cardiovascular disease, chronic kidney disease, chronic liver disease, and smoking who presented with dyspnea and shortness of breath. Portable CXR shows left lung opacities with a total Sextus score = 3 with opacities in the entire right lung. (C) CXR of a 61-year old overweight man (BMI = 27) with a history of hypertension and hyperlipidemia who presented with persistent dry cough. CXR shows diffuse lung opacities in bilateral lungs with a total Sextus score = 6. BMI = body mass index.

A 2-step 6-zonal assessment (e.g., “Sextus score”) was calculated on both CXRs and coronal chest CT sections (Fig. 2). First, each lung was divided into 3 zones: upper (above the carina), middle (below the carina up to the inferior pulmonary vein), and lower (below the inferior pulmonary vein). When anatomical landmarks were unable to be adequately identified, each lung was divided into 3 equal zones. Second, each zone was assigned a binary score based on the absence (0) or presence (1) of opacities at least 1 cm^2^ in size. The points of each lung zone were summed together (minimum score 0, maximum score 6).

### 2.5 Validation study

All CXR and CT images were evaluated independently by 2 naïve readers (JS, KY) trained and supervised by a fellowship-trained cardiothoracic radiologist with 10 years’ experience (HSM). CXR and CT images which were acquired within 24 hours of each other without any intervening interventional measures (e.g., intubation or tracheostomy) were assessed for correlation. A random subset of 1 CXR from each of the 124 patients and 30 CTs were independently scored by a second reader (KY) to test for reproducibility. After which, all scores were agreed by consensus by a third-party arbiter (HSM) resolving any discrepant scores. To minimize bias, all readers were blinded to patient histories other than COVID-19 positivity. Readers were blinded to radiologic report as well as each other's scoring, which was performed on 2 separate workstations not linked to picture archive and communications system.

### 2.6 Statistical analysis

Descriptive summary data were expressed as counts and percentages for categorical variables and median (interquartile range [IQR] or range) for continuous variables. Comparisons between 2 groups were performed using Fisher exact or chi-square test for categorical variables and Wilcoxon rank test for continuous variables. Associations between outcomes such as intubation, prolonged length of hospitalization, or mortality and potential risk factors were first evaluated using raw/unadjusted odds ratios and its 95% confidence intervals (CIs) via univariable logistic regression, with *P* values from the Wald test in the regression model. Multivariable logistic regression models were used to examine the associations of individual potential risk factors of interest with each of the clinical outcome variables (except for mortality due to its overall low event rate) 1 at a time while adjusting for other potential confounding factors or effect-modifiers. Variable selection was implemented in a step-wise fashion. Adjusted odds ratios (aORs) and their 95% CIs based on these regression models were reported as appropriate. The agreement or inter-rater reliability between CXR Sextus scores from the 2 readers was examined using the weighted kappa and its 95% CI. CXR Sextus scores were correlated with CT Sextus scores and other lab parameters using Spearman correlation coefficient (SCC). Two-tailed *P* values of less than .05 were considered statistically significant. SAS version 9.4 (SAS Institute, Carey, NC) was used to carry out all the data analyses.

## 3 Results

A total of 124 patients were included (median [IQR] age 58.5 [47.5–69.0] years) with additional demographic data represented in Table 1. There were 3 lung transplant patients; 2 with a history of lung transplant and 1 who was diagnosed with COVID-19 during posttransplant recovery. Eighty-four (68%) patients tested positive and 37 (30%) negative on their initial RT-PCR test, and 3 (2%) did not have any documented RT-PCR tests during their admission. Of the 124 chest CT scans, 107 (86%) were category 1 and 17 (14%) category 2. One patient passed away in the emergency department before being transported to an isolation ward, 86 (69%) patients were cared for in isolation wards, and 38 (31%) patients were admitted to the ICU at least once during their admission. The overall median length of hospital stay was 6 [IQR 3–13] days (Table 1).

**Table 1:** Patient demographics and clinical findings in ICU and ward settings.

Variables	All patients (n = 124)	ICU patients (n = 38)	Ward patients (n = 86)
Age, median [IQR] (yrs)	58.5 [47.5–69.0]	63.0 [54.0–74.0]	55.5 [45.0–68.0]
Sex (male, N (%))	72 (58)	15 (40)	57 (66)
Race/ethnicity (N (%))			
White	18 (15)	8 (21)	10 (12)
Black	58 (47)	21 (55)	37 (43)
Hispanic	35 (28)	8 (21)	27 (31)
Asian	3 (2)	1 (3)	2 (2)
Other/unknown	10 (8)	0 (0)	10 (12)
Smoking history (N (%))^∗^			
Never	66 (53)	17 (45)	49 (57)
Current	20 (16)	5 (13)	15 (18)
Former	34 (27)	15 (40)	19 (22)
Unknown	4 (3)	1 (3)	3 (3)
BMI, median [IQR] (kg/m^2^)^∗^	30.9 [25.6–36.5]	30.4 [23.8–36.9]	31.2 [26.4–35.9]
BMI cutoffs (kg/m^2^) (N (%))^∗^			
Non-obese (≤30)	57 (46)	18 (47)	39 (46)
Obese (>30)	65 (52)	20 (53)	45 (54)
Comorbidities (N (%))^∗^			
HTN	75 (61)	23 (61)	52 (61)
T2DM	50 (41)	19 (50)	31 (37)
Asthma	22 (18)	6 (16)	16 (19)
COPD	18 (15)	8 (21)	10 (12)
Malignancy	16 (13)	8 (21)	8 (9)
Cardiovascular disease	18 (15)	6 (16)	12 (14)
Cerebrovascular disease	6 (5)	2 (5)	4 (5)
CKD	18 (15)	8 (21)	10 (12)
HLD	39 (32)	18 (47)	21 (25)
HIV	4 (3)	1 (3)	3 (4)
Procedures (N (%))			
Intubation	24 (19)	23 (61)	1^†^ (1)
Duration on ventilator, median [IQR] (d)	–	4 [0–11]	–
Tracheostomy	6 (5)	6 (16)	0 (0)
Length of hospitalization (d)			
Total length, median [IQR]	6.0 [3.0–13.0]	14.0 [8.0–19.0]	4.5 [3.0–7.0]
ICU length of stay, median [IQR]	–	8.0 [4.0–16.0]	–
RT-PCR results (N (%))^∗^			
Negative	37 (31)	11 (30)	26 (31)
Positive	84 (69)	26 (70)	58 (69)
Mortality (N (%))	13 (10)	12 (32)	1^∗^ (1)

Categorical variables are expressed as counts and percentages. Continuous variables are expressed as medians with interquartile ranges [IQR]. BMI = body mass index, CKD = chronic kidney disease, COPD = chronic obstructive pulmonary disease, HIV = human immunodeficiency virus, HLD = hyperlipidemia, HTN = hypertension, ICU = intensive care unit, RT-PCR = reverse transcriptase-polymerase chain reaction, T2DM = type II diabetes mellitus.

∗ 4, 2, 1, 3 patients were missing smoking history, BMI, comorbidity, and RT-PCR data respectively.

† Patient passed away in emergency department before transport to isolation ward.

### 3.1 Sextus CXR scoring validation

**Figure 3:**
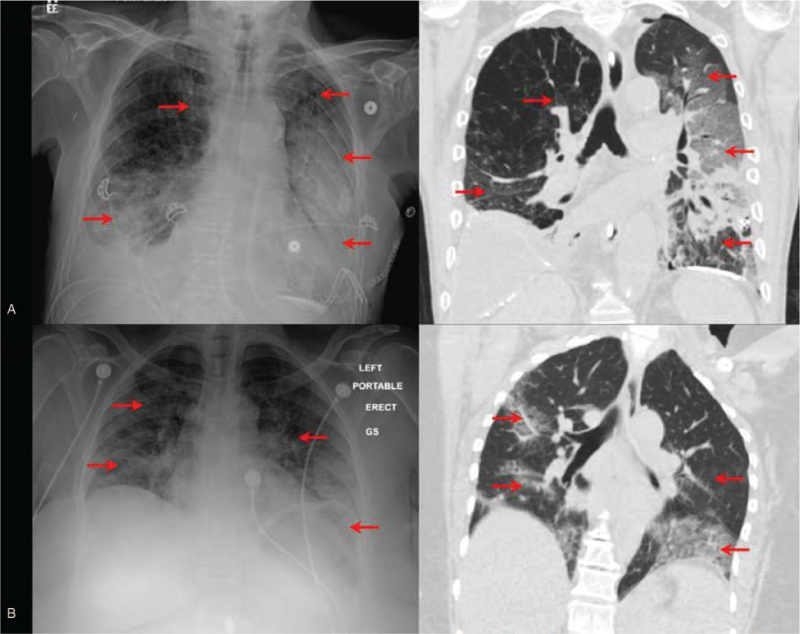
Examples of chest radiography (CXR) and chest computed tomography (CT) correlations. (A) CXR and CT images of a 75-year old man with a history of chronic obstructive pulmonary disease, hyperlipidemia, and smoking who was found COVID-19 positive on nasal swab testing. CXR and CT show opacities in the right upper and lower lobes and entire left lung with a total Sextus scores of 5. (B) CXR and CT images of a 63-year old obese woman (BMI = 37) with a history of smoking who presented with dry cough, dyspnea, and shortness of breath. CXR and CT show opacities in the middle and lower lobes of bilateral lungs with a total Sextus scores of 4. COVID-19 = coronavirus disease 2019. BMI = body mass index.

CXR scores at presentation ranged from 1 to 6 (median 4, [IQR 2–5]). CT scores at presentation ranged from 1 to 6 (median 4, [IQR 2.5–5]). CXRs were scored independently by 2 readers with a weighted kappa of 0.76 (95% CI: 0.69–0.83), indicating a substantial inter-rater reliability. A total of 116 CXR and CT pairs were identified with a SCC = 0.75, *P* < .0001 (Fig. 3), implying a highly positive association between CXR and CT scores.

### 3.2 Secondary outcomes of CXR Sextus score

Demographics and clinical findings in relation to the presenting CXR Sextus score (≥3 and ≥5) are presented for all 124 patients (Table 2). The severity (≥3) of the presenting CXR scores was not significantly associated with age, smoking status, sex, or BMI. Care level and CT Sextus score upon presentation were somewhat significantly associated with a high CXR Sextus score (≥3) with a *P* value of 0.08 and <.0001, respectively.

**Table 2:** Patient demographics and clinical findings in relation to CXR Sextus score at presentation for 124 admitted patients.

Variables	All patients (n = 124)	Low CXR Sextus score 0–2^†^ (n = 33)	High CXR Sextus score 3–6^†^ (n = 91)	*P* value	Mild and moderate CXR Sextus score 0–4 (n = 87)	Severe CXR Sextus score 5–6 (n = 37)	*P* value
Age, median [IQR] (yrs)	58.5 [47.5–69.0]	58 [46.0–69.0]	59 [48.0–68.0]	.99	58 [46.0–69.0]	61.0 [49.0–65.0]	.85
Sex (male, N (%))	72 (58)	20 (61)	52 (57)	.84	55 (63)	17 (46)	.11
Race/ethnicity (N (%))				**.045**			.15
Black	58 (47)	18 (55)	40 (44)		41 (47)	17 (46)	
Hispanic	35 (28)	12 (36)	23 (25)		28 (32)	7 (19)	
White/Asian/Other	31 (25)	3 (9)	28 (31)		18 (21)	13 (35)	
Smoking history (N (%))^∗^				.51			.44
Never	66 (53)	15 (47)	51 (58)		47 (55)	19 (54)	
Current smoker	20 (15)	7 (22)	13 (15)		12 (14)	8 (23)	
Former smoker	34 (27)	10 (31)	24 (27)		26 (31)	8 (23)	
BMI, median [IQR] (kg/m^2^)^∗^	30.9 [25.6–36.5]	31.3 [25.9–33.3]	30.6 [25.6–36.9]	.47	30.2 [25.2–35.9]	32.6 [26.8–37.4]	.060
BMI cutoffs (kg/m^2^) (N (%))^∗^				.84			.32
Non-obese (≤30)	57 (47)	14 (44)	43 (48)		43 (50)	14 (39)	
Obese (>30)	65 (53)	18 (56)	47 (52)		43 (50)	22 (61)	
Comorbidities (N (%))^∗^							
HTN	75 (61)	19 (58)	56 (62)	.68	56 (64)	19 (53)	.31
T2DM	50 (41)	15 (46)	35 (39)	.54	35 (40)	15 (42)	1.00
Asthma	22 (18)	6 (18)	16 (18)	1.00	17 (20)	5 (14)	.61
COPD	18 (15)	4 (12)	14 (16)	.78	13 (15)	5 (14)	1.00
Malignancy	16 (13)	2 (6)	14 (16)	.23	10 (12)	6 (17)	.56
Cardiovascular disease	18 (15)	6 (18)	12 (13)	.57	12 (14)	6 (17)	.78
CKD	18 (15)	7 (21)	11 (12)	.25	14 (16)	4 (11)	.58
HLD	39 (32)	12 (36)	27 (30)	.52	29 (33)	10 (28)	.67
Care level (N (%))				.08			**<.0001**
ICU	38 (31)	6 (18)	32 (35)		17 (20)	21 (57)	
Ward	86 (69)	27 (82)	59 (65)		70 (80)	16 (43)	
RT-PCR results (N (%))^∗^				.83			.52
Negative	37 (31)	11 (33)	26 (30)		24 (29)	13 (35)	
Positive	84 (69)	22 (67)	62 (71)		60 (71)	24 (65)	
Mortality (N (%))	13 (10)	2 (6)	11 (12)	.51	5 (6)	8 (22)	.020
First CT Sextus Score (0–6), median [IQR]	4.0 (2.5–5.0)	2.0 (2.0–3.0)	5.0 (3.0–6.0)	**<.0001**	3.0 (2.0–4.0)	6.0 (5.0–6.0)	**<.0001**

Categorical variables are expressed as counts and percentages. Continuous variables are expressed as medians with interquartile ranges [IQR]. Significant *P* values (<.05) are bolded. BMI = body mass index, CKD = chronic kidney disease, COPD = chronic obstructive pulmonary disease, CT = computed tomography, CXR = chest radiography, HLD = hyperlipidemia, HTN = hypertension, ICU = intensive care unit, RT-PCR = reverse transcriptase-polymerase chain reaction, T2DM = type II diabetes mellitus.

∗ 4, 2, 1, 3 patients were missing smoking history, BMI, comorbidity, and RT-PCR data respectively.

† Toussei et al investigated patient demographics and clinical variables in relation to low (0–2) and high (3–6) CXR severity scores.

Of the 124 patients, 24 (19%) were intubated, 43 (35%) had a prolonged hospitalization (≥10 days), and 13 (10%) died from COVID-19-related illness. The overall mortality rate was 10.5% with a median age of 72.0 [IQR 62.0–75.0] years for the deceased compared to 57.0 [IQR 46.0–68.0] years for the survivors. Nine (69%) of the deceased patients were male.

**Figure 4:**
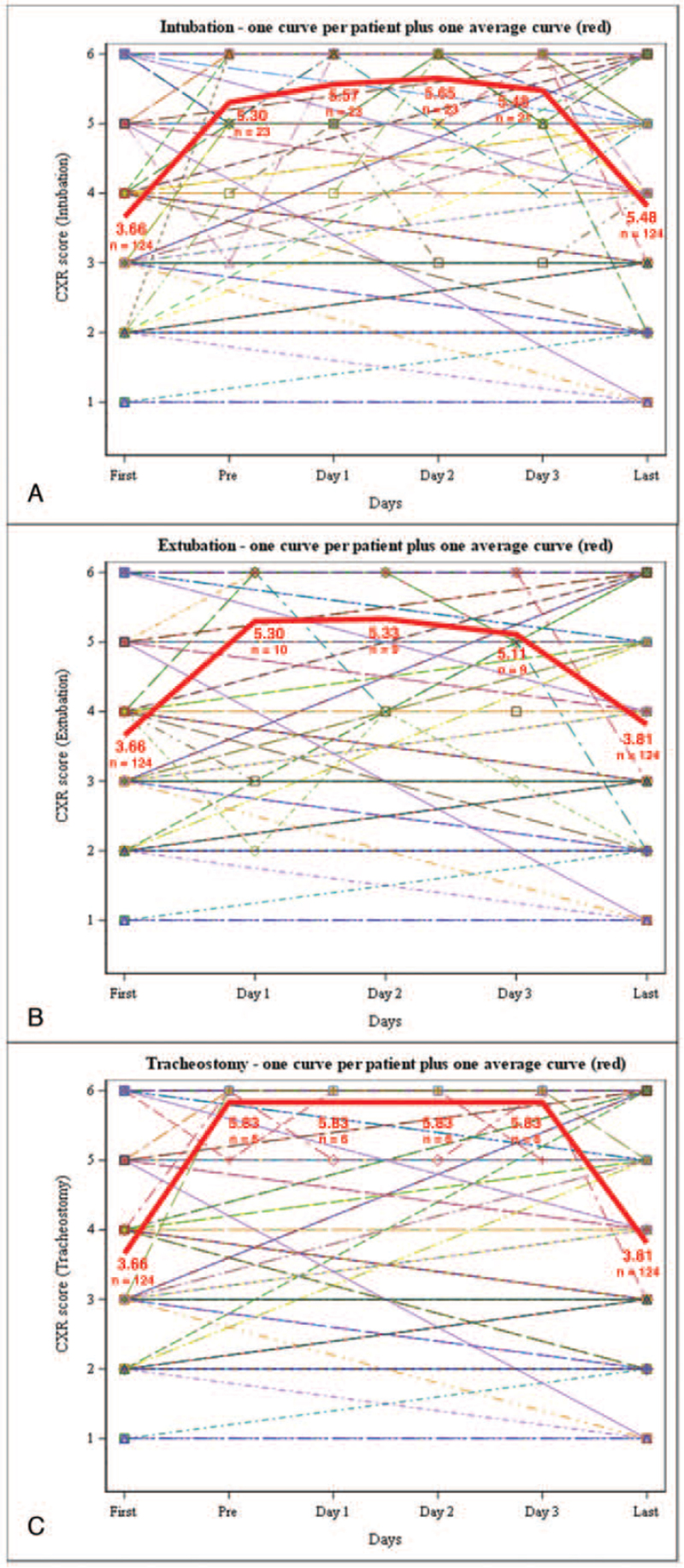
Chest radiography (CXR) Sextus scores (A) peri-intubation, (B) peri-extubation, and (C) peri-tracheostomy. Solid thick red line represents the mean CXR Sextus scores of all patients. First = first CXR Sextus score; Pre = CXR Sextus score pre-procedure (intubation, tracheostomy); Day 1, 2, 3 = CXR Sextus score the first 3 days postprocedure (intubation, tracheostomy, extubation); Last = final CXR Sextus score before endpoint of discharge/AMA or death.

The median changes between presenting CXR Sextus scores and first intubation, first tracheostomy, or final CXR Sextus scores for the entire cohort were 1.0 (n = 23), 1.0 (n = 6), and 0.0 (n = 124), respectively (see Table S1, Supplemental Digital Content, http://links.lww.com/MD2/A642, which illustrates the median Sextus score changes in relation to patients’ admission). We also observed mean CXR scores slightly increasing after intubation, decreasing after extubation, and remaining stable after tracheostomy (Fig. 4). CXR Sextus scores and lactate dehydrogenase values were significantly correlated during the first 3 days of admission, with a SCC of 0.48 (*P* = .046, n = 18), 0.68 (*P* < .001, n = 38), and 0.69 (*P* < .001, n = 53), respectively. There was generally a weak correlation between the CXR score and leukocyte count values (SCC = 0.22, *P* = .027, n = 104; SCC = 0.31, n = 40; *P* = 0.025, n = 52; SCC = 0.15, *P* = 0.20, n = 77, respectively) and between CXR score and C-reactive protein values (SCC = 0.43, *P* = .068, n = 19; SCC = 0.48, *P* = .002, n = 40; SCC = 0.20, *P* = .14, n = 56, respectively) for the first 3 days of admission.

### 3.3 CXR Sextus scores

The initial CXR Sextus score was significantly associated with adverse outcomes in unadjusted as well as adjusted data analyses. Incremental increases of CXR Sextus scores of 2 points were an independent predictor of intubation (aOR: 2.12, 95% CI 1.41–3.19, *P* = .0003), and prolonged hospitalization (≥10 days) (aOR: 2.01, 95% CI 1.41–2.88, *P* = .0001) (Table 3). A CXR Sextus score ≥3 was an independent predictor of intubation (aOR: 6.54, 95% CI 1.28–33.47, *P* = .024) and prolonged hospitalization (aOR: 3.95, 95% CI 1.30–12.01, *P* = .016). Individual clinical predictors of intubation included age, chronic obstructive pulmonary disease, chronic kidney disease, and presenting CT Sextus score (odds ratio [OR]: 1.59, 95% CI 1.15–2.19, *P* = .005) in addition to the CXR Sextus score (OR: 3.45, 95% CI 1.71–6.97 for 2 points higher of CXR score). There were no statistically significant differences in intubation rates between race, sex, BMI, asthma, smoking status, or T2DM groups. Individual clinical predictors of a prolonged length of hospitalization included age, race, T2DM, smoking status, and presenting CT score (OR: 1.44, 95% CI 1.12–1.85, *P* = .005) in addition to the CXR Sextus score (OR: 2.20, 95% CI 1.29–3.75 for 2 points higher of CXR score). In univariable analysis, incrementally increasing CXR Sextus score by 2 points was found to be significantly associated with mortality (OR: 2.31, 95% CI 1.01–5.29, *P* = .048). Furthermore, a presenting CXR score ≥5 was found to be a significant predictor of mortality (OR: 4.52, 95% CI 1.37–14.94, *P* = .020). Individual clinical predictors of mortality included age and smoking status (see Table S2, Supplemental Digital Content, http://links.lww.com/MD2/A643, which illustrates patient demographics, clinical findings, and presenting CXR scores in relation to outcomes of interest).

**Table 3:** The relationship between clinical factors and presenting CXR Sextus score for risk of mortality, intubation, and length of hospitalization (n = 124)^∗^.

	Intubation (19%, 23/124)			Prolonged length of hospitalization ≥ 10 days (35%, 43/124)			Mortality (10%, 13/124)^‡^
Variables	Unadjusted odds ratio (95% CI)	Adjusted odds ratio (95% CI) for CXR Sextus score ≥ 3	Adjusted odds ratio (95% CI) for CXR Sextus score (0–6)	Unadjusted odds ratio (95% CI)	Adjusted odds ratio (95% CI) for CXR Sextus score ≥ 3	Adjusted odds ratio (95% CI) for CXR Sextus score (0–6)	Unadjusted odds ratio (95% CI)
Age (for increment = 11 years)	**1.64 (1.11–2.43)**	1.04–1.00–1.08	**1.05 (1.00–1.09)**	**1.63 (1.18–2.23)**	**1.05 (1.02–1.08)**	**1.06 (1.02–1.10)**	**2.02 (1.15–3.54)**
Sex (reference: male)	1.50 (0.61–3.67)	–	–	2.06 (0.97–4.37)	–	–	0.58 (0.17–2.00)
Race/ethnicity							
Black	Reference	–	–	Reference	Reference	Reference	Reference
Hispanic	0.45 (0.13–1.50)	–	–	**0.40 (0.16–0.99)**	0.31 (0.10–0.94)	**0.29 (0.09–0.91)**	1.12 (0.29–4.28)
White/Asian/Other	1.01 (0.36–2.87)	–	–	**0.33 (0.12–0.90)**	0.42 (0.13–1.40)	**0.26 (0.08–0.89)**	0.93 (0.22–4.00)
Smoking history^†^							
Never	Reference	–	–	Reference	Reference	Reference	Reference
Current smoker	1.81 (0.48–6.80)	–	–	**1.14 (0.38–3.43)**	–	–	Not estimatable^§^
Former smoker	3.02 (1.06–8.58)	–	–	**3.00 (1.26–7.12)**	–	–	**4.02 (1.09–14.88)**
BMI (for increment = 5 kg/m^2^)^†^	0.85 (0.62–1.16)	–	–	0.98 (0.77–1.24)	–	–	0.81 (0.53–1.24)
BMI cutoffs (kg/m^2^)^†^							
Non-obese (≤30)	Reference	–	–	Reference	–	–	Reference
Obese (>30)	0.76 (0.31–1.89)	–	–	1.01 (0.48–2.13)	–	–	0.86 (0.26–2.86)
Comorbidities^†^							
HTN	1.25 (0.48–3.22)	–	–	1.79 (0.82–3.94)	–	–	0.88 (0.26–2.94)
T2DM	2.21 (0.88–5.55)	–	–	**2.62 (1.22–5.62)**	–	–	2.21 (0.66–7.42)
Asthma	0.64 (0.17–2.38)	–	–	0.65 (0.23–1.82)	–	–	0.39 (0.05–3.23)
COPD	**3.54 (1.19–10.50)**	2.61 (0.78–8.73)	2.98 (0.79–11.25)	1.22 (0.44–3.42)	–	–	2.13 (0.52–8.79)
Malignancy	3.18 (1.02–9.90)	–	–	2.06 (0.71–5.94)	–	–	2.51 (0.60–10.49)
Cardiovascular disease	1.86 (0.59–5.87)	–	–	1.60 (0.58–4.41)	–	–	3.46 (0.92–13.03)
CKD	**3.54 (1.19–10.50)**	**3.91 (1.12–13.61)**	**4.42 (1.16–16.87)**	2.73 (0.99–7.54)	3.08 (0.93–10.16)	**3.53 (1.02–12.19)**	3.46 (0.92–13.03)
HLD	1.88 (0.74–4.78)	–	–	2.02 (0.92–4.43)	–	–	2.36 (0.71–7.87)
RT-PCR results (N (%))^†^							
Negative	Reference	–	–	Reference	–	–	Reference
Positive	1.09 (0.41–2.90)	–	–	**3.06 (1.21–7.76)**	**3.40 (1.16–10.03)**	**4.64 (1.46–14.81)**	1.53 (0.40–5.92)
Presenting CXR Sextus score (0–6) (for increment = 2)	**3.45 (1.71–6.97)**	–	**2.12 (1.41–3.19)**	**2.20 (1.29–3.75)**	–	**2.01 (1.41–2.88)**	**2.31 (1.01–5.29)**
Presenting CXR Sextus score ≥ 3	**4.94 (1.09–22.33)**	**6.54 (1.28–33.47)**	–	2.43 (0.96–6.19)	**3.95 (130–12.01)**	–	2.13 (0.45, 10.17)
Presenting CXR Sextus score ≥ 5	**4.69 (1.84–11.95)**	–	–	**2.77 (1.25–6.15)**	–	–	**4.52 (1.37–14.94)**
First CT Sextus score (0–6) (for increment = 1)	**1.59 (1.15–2.19)**	–	–	**1.44 (1.12–1.85)**	–	–	1.44 (0.96–2.14)

BMI = body mass index, CI = confidence interval, CKD = chronic kidney disease, COPD = chronic obstructive pulmonary disease, CT = computed tomography, CXR = chest radiography, HLD = hyperlipidemia, HTN = hypertension, RT-PCR = reverse transcriptase-polymerase chain reaction, T2DM = type II diabetes mellitus.

∗ Data in parenthesis are 95% confidence intervals. Significant *P* values (<.05) are bolded. Adjusted odds ratios (95% CIs) were calculated from a final logistic regression model that included only (near) significant covariates at *P* < .10 via a model selection procedure for each outcome.

† 4, 2, 1, 3 patients were missing smoking history, BMI, comorbidity, and RT-PCR data, respectively.

‡ Unable to perform multiple logistic regression for adjusted odds ratios due to low event count of mortality (n = 13).

§ Unable to estimate the odds ratio for this category due to 0 event in this category.

## 4 Discussion

While chest CT has earned its role as the reference standard for COVID-19 imaging,^[20]^ there is clearly a demand for improved understanding of CXR capacity in the imaging of a COVID-19 positive patient. As the COVID-19 pandemic continues to strain healthcare systems globally, the need for a quick and objective CXR scoring algorithm – to assist the flow of triage and management as well as prognosticate outcomes – becomes even more imperative. Our analysis of the Sextus score has shown that it is comparable to CT in both the triage and management of COVID-19 patients (CXR-CT severity score kappa = 0.75, *P* < .0001). Another advantage of this Sextus scoring system is its simplicity and reproducibility, as 2 naïve readers were able to learn to interpret and score with high fidelity, achieving a tight inter-reader agreement of CXR Sextus scores (inter-rater kappa = 0.76, 95% CI 0.69–0.83). CXR Sextus score may also be used as a prognosticator^[20]^ as higher CXR Sextus scores were associated with higher mortality (OR: 2.20, 95% CI 1.29–3.75 for 2 points higher of CXR score) as well as an independent predictor of intubation and prolonged hospitalization (≥10 days) especially when scores were ≥3.

The composition of our patient cohort reflects the ethnic and socio-economic tapestry of the urban inner-city North Philadelphia community.^[21]^ Underserved populations and minorities often experience disproportionate disease burden and adverse outcomes, and COVID-19 is no exception.^[22–24]^ This disparity is demonstrated in the degree of severity in non-white COVID-19 patient CXRs.^[23]^ Our investigation purports that Black ethnicity is associated with prolonged length of hospitalization for COVID-19. With an overall median length of hospitalization of 6 days and discharge rate of 89.5% throughout all ethnicities, identifying and triaging high-risk patients is crucial to effectively utilizing limited resources.

The authors developed this CXR Sextus scoring system concurrently but independently from the similar CXR severity scoring system developed by Toussie et al.^[17]^ Both scoring systems share comparable features with the primary difference being the added objective measure of opacities (1 cm^2^ vs undefined) and the application of the Sextus scoring system to both CXRs and CTs. However, our selection criteria of COVID-19 positive patients with positive imaging findings only allowed the analysis of a unique cohort of COVID-19, as positive imaging findings have showed a higher sensitivity compared to that of RT-PCR testing.^[8,25]^ Our findings also parallel that of the similar system developed by Borghesi^[14]^ but also provide additional insight into comparisons between the CXR severity score and patient comorbidities. Despite different patient populations, our results not only support their findings that CXR severity scoring is reliable and may be a useful tool in the hand of the clinician, but also strengthen Toussie et al's claim that CXR can be an independent prognostic predictor of outcomes such as intubation and prolonged length of hospitalization (also defined as ≥10 days).

Our results are congruent with the World Health Organization's recent Rapid Advice Guide suggesting the use of chest imaging including CXR to inform therapeutic management in symptomatic hospitalized COVID-19 patients.^[26]^ However, these recommendations address chest imaging as a whole and do not address the indications or frequency of specific imaging modalities. Chest CT is much more sensitive and specific for pulmonary sequelae of COVID-19, with sensitivities ranging from 86% to 96%,^[6,8,25]^ while CXR sensitivity and specificity are reported as 57% and 89%, respectively.^[13,25]^ However, logistical factors such as room turnover, limited equipment, radiation dose, and time to transport patients limit the widespread use of CT.^[27,28]^ The streamlined processes for portable CXR implementation lend themselves to greater accessibility. Additionally, CXR can provide advantages over CT in directing patient care in real time. Using the Sextus score, daily chest radiographs can provide clinicians with data on which patients are at higher risk for requiring intubation independent of other clinical factors. As no clear guidelines existed for COVID-19 imaging, our team found that the combination of a single chest CT upon presentation, if appropriately indicated, followed by serial CXRs may help understanding and monitoring of the disease course.

Ultimately, there remains a continued need for clear and robust diagnostic parameters for both CT and CXR imaging, both independently and collectively, in the management of COVID-19 patients. Our analyses demonstrate the reliability and simplicity of the CXR Sextus score and offers an approach to more objectively interpret patients’ CXR (see Figure S1, Supplemental Digital Content, http://links.lww.com/MD2/A641, which illustrates patient series of daily CXR Sextus scores). Serial CXRs may as well have a role in continually monitoring COVID-19 disease progression, complications, and patient response to treatment allowing further optimization of patient management. Our observations of increasing CXR Sextus scores peri-intubation and decreasing CXR Sextus scores peri-extubation provide insight into when patients may be at higher risk for adverse outcomes.

Although our sample size is similar to that of Toussei et al, the primary limitation of our study is its limited sample size and single network nature. In our study, 13 (10%) patients died during the study period, a subgroup too small to permit evaluation of independent statistical associations of potential risk factors with mortality. A multicenter study using the same Sextus score would provide a more robust insight into potential trends and associations of this disease morbidity and mortality. Additional limitations are that a mixture of both anterior-posterior and posterior-anterior views were used to calculate Sextus severity scores and its lack of translatability to patients with fewer than 6 lung zones. Additionally, several patients with multiple admissions during this time period were considered to have a single combined admission which could have affected the overall length of hospitalization.

## 5 Conclusion

The CXR Sextus score was shown to be reproducible and quite comparable to the reference standard CT, for COVID-19 imaging. Sextus score was also predictive of risks for intubation and prolonged hospitalization for COVID-19 patients in a predominantly Black population. A simplistic and reproducible objective CXR scoring system may offer important insights into management of COVID-19 patients. Further multicenter evaluation of the CXR Sextus score may provide added insight into identifying high-risk patients, additional clinical predictors of outcome, and hopefully build upon improving COVID-19 triaging in similar high-volume scenarios. Future studies of the CXR Sextus score may also allow us to better determine the optimal frequency of CXR use in hospitalized COVID-19 patients, especially in minority populations.

## Acknowledgments

The authors would like to thank all the front-line responders and their continued efforts during these tumultuous times.

## Author contributions

JS is responsible for study concept, data extraction, image scoring, analysis of data, table and figure creation, and manuscript preparation. DY is responsible for analysis of data, table and figure creation, and manuscript editions. KY is responsible for data extraction, image scoring, and manuscript editions. RC is responsible for data extraction and manuscript editions. XL is responsible for analysis of data. TS, GC, NM, OA, MK are responsible for manuscript editions. HM is the guarantor of integrity of the study and is responsible for study concept, image scoring, analysis of data, and manuscript preparation. All authors read and approved the final manuscript.

**Conceptualization:** Justin Sun, Gary Cohen, Nathaniel Marchetti, Maruti Kumaran, Hillel Maresky.

**Data curation:** Justin Sun, Kevin Yoo, Robert Choi, Hillel Maresky.

**Formal analysis:** Justin Sun, Daohai Yu, Xiaoning Lu, Omar Agosto, Hillel Maresky.

**Investigation:** Justin Sun, Kevin Yoo, Nathaniel Marchetti, Omar Agosto, Hillel Maresky.

**Methodology:** Justin Sun, Daohai Yu, Kevin Yoo, Xiaoning Lu, Taylor Standiford, Gary Cohen, Nathaniel Marchetti, Omar Agosto, Maruti Kumaran, Hillel Maresky.

**Project administration:** Taylor Standiford, Gary Cohen, Maruti Kumaran, Hillel Maresky.

**Resources:** Justin Sun, Omar Agosto, Maruti Kumaran, Hillel Maresky.

**Software:** Daohai Yu.

**Supervision:** Taylor Standiford, Gary Cohen, Nathaniel Marchetti, Omar Agosto.

**Validation:** Justin Sun, Daohai Yu.

**Visualization:** Justin Sun, Daohai Yu, Xiaoning Lu, Hillel Maresky.

**Writing – original draft:** Justin Sun, Daohai Yu, Kevin Yoo, Robert Choi, Xiaoning Lu, Taylor Standiford, Gary Cohen, Nathaniel Marchetti, Omar Agosto, Maruti Kumaran, Hillel Maresky.

**Writing – review & editing:** Justin Sun, Daohai Yu, Kevin Yoo, Robert Choi, Xiaoning Lu, Taylor Standiford, Gary Cohen, Nathaniel Marchetti, Omar Agosto, Maruti Kumaran, Hillel Maresky.

## Supplementary Material

SUPPLEMENTARY MATERIAL
