# Supplementary material for: Sextus chest radiograph severity score correlates to clinical outcomes in patients with COVID-19: A cross-sectional study
Source: Medicine (Baltimore). 2021 Nov 12;100(45):e27663. doi: 10.1097/MD.0000000000027663 (PMC10545010; doi:10.1097/MD.0000000000027663)
Supplement: SUPPLEMENTARY MATERIAL [file medi-100-e27663-s001.doc]

**Figure S1 — Snapshot of daily CXR Sextus scores from days 1 to 5.**

***A)* 57-year old obese man (BMI=34) with a history of hypertension, malignancy, and hyperlipidemia who presented with dyspnea and shortness of breath with daily CXR Sextus scores of 4, 6, 6, 4, 5 over 7 days of hospitalization. *B)* 53-year old overweight man (BMI=27) with a history of type II diabetes mellitus and chronic obstructive pulmonary disease who presented with fever and expectoration with daily CXR Sextus scores of 5, 3, 3, 2, 3 over 5 days of hospitalization.**

**Supplemental Digital Content 3. Pictorial series of CXR Sextus scores illustrating series of daily CXR Sextus scores. tiff**
