# Supplementary material for: Sextus chest radiograph severity score correlates to clinical outcomes in patients with COVID-19: A cross-sectional study
Source: Medicine (Baltimore). 2021 Nov 12;100(45):e27663. doi: 10.1097/MD.0000000000027663 (PMC10545010; doi:10.1097/MD.0000000000027663)
Supplement: SUPPLEMENTARY MATERIAL [file medi-100-e27663-s002.doc]

**Table S1: Median and [IQR/Range] of Sextus score changes in relation to patients’ admission**

| **Variable** | **Overall (n=124)** | **Discharge/AMA (n=111)** | **Death (n=13)** |
| --- | --- | --- | --- |
| **CXR Sextus Score Overall Change (Last – First), n** | 124 | 111 | 13 |
| **Median [IQR]** | 0.0 (-1.0, 1.0) | 0.0 (-1.0, 1.0) | 1.0 (-1.0, 1.0) |
| **Median [Range]** | 0.0 (-4.0, 4.0) | 0.0 (-4.0, 4.0) | 1.0 (-1.0, 4.0) |
| **CT Sextus Score Overall Chagne (Last – First), n** | 19 | 16 | 3 |
| **Median [IQR]** | 0.0 (0.0, 2.0) | 0.0 (0.0, 2.5) | 1.0 (0.0, 2.0) |
| **Median [Range]** | 0.0 (-2.0, 4.0) | 0.0 (-2.0, 4.0) | 1.0 (0.0, 2.0) |
| **Intubation – First, n** | 23 | 11 | 12 |
| **Median [IQR]** | 1.0 (0.0, 2.0) | 1.0 (0.0, 2.0) | 1.0 (0.5, 2.0) |
| **Median [Range]** | 1.0 (-1.0, 4.0) | 1.0 (-1.0, 2.0) | 1.0 (-1.0, 4.0) |
| **Extubation – First, n** | 10 | 8 | 2† |
| **Median [IQR]** | 0.0 (0.0, 2.0) | 0.0 (0.0, 2.0) | -1.0, 1.0 |
| **Median [Range]** | 0.0 (-2.0, 2.0) | 0.0 (-2.0, 2.0) | -1.0, 1.0 |
| **Tracheostomy – First, n** | 6 | 5 | 1† |
| **Median [IQR]** | 1.0 (0.0, 1.0) | 1.0 (0.0, 1.0) | 1.0 |
| **Median [Range]** | 1.0 (0.0, 3.0) | 1.0 (0.0, 3.0) | 1.0 |

**AMA=against medical advice; CXR=chest radiography; CT=computed tomography; First=first CXR taken upon arrival to hospital; Last=last CXR taken before endpoint; Intubation=first CXR after Intubation; Extubation=first CXR after extubation; Tracheostomy=first CXR after placement of tracheostomy tube**

**†Not enough data points, only raw data were listed here.**

**Supplemental Digital Content 1. Table that illustrates the median Sextus score changes in relation to patients’ admission. docx**
