# Supplementary material for: Sextus chest radiograph severity score correlates to clinical outcomes in patients with COVID-19: A cross-sectional study
Source: Medicine (Baltimore). 2021 Nov 12;100(45):e27663. doi: 10.1097/MD.0000000000027663 (PMC10545010; doi:10.1097/MD.0000000000027663)
Supplement: SUPPLEMENTARY MATERIAL [file medi-100-e27663-s003.doc]

**Table S2: Patient demographics, clinical findings, and presenting CXR scores for 124 patients in the inpatient setting in relation to outcomes of interest including mortality, intubation, and prolonged length of hospitalization**

|  |  | **OUTCOMES** | | | | | | | | |
| --- | --- | --- | --- | --- | --- | --- | --- | --- | --- | --- |
| **Variables** | **All Patients (n=124)** | **Discharge/AMA (n=111)** | **Death (n=13)** | **P-value** | **Not Intubated (n=100)** | **Intubated (n=24)** | **P-value** | **Length of Hospitalization <10 days (n=81)** | **Length of Hospitalization ≥10 days (n=43)** | **P-value** |
| **Age, median [IQR] (years)** | 58.5 [47.5-69.0] | 57 [46.0-68.0] | 72 [62.0-75.0] | **0.014** | 56 [46.0-67.5] | 63.5 [57.0-76.0] | **0.013** | 54 [46.0-65.0] | 63 [56.0-75.0] | **0.003** |
| **Sex (Male, N (%))** | 72 (58) | 63 (57) | 9 (69) | 0.56 | 60 (60) | 12 (50) | 0.49 | 52 (64) | 20 (46) | 0.09 |
| **Race/ethnicity (N (%))** |  |  |  | 0.97 |  |  | 0.38 |  |  | **0.032** |
| **Black** | 58 (47) | 52 (47) | 6 (46) |  | 45 (45) | 13 (54) |  | 31 (38) | 27 (63) |  |
| **Hispanic** | 35 (28) | 31 (28) | 4 (31) |  | 31 (31) | 4 (17) |  | 26 (32) | 9 (21) |  |
| **White/Asian/Other** | 31 (25) | 28 (25) | 3 (23) |  | 24 (24) | 7 (29) |  | 24 (30) | 7 (16) |  |
| **Smoking History (N (%))*** |  |  |  | **0.017** |  |  | 0.10 |  |  | **0.034** |
| **Never** | 66 (53) | 62 (56) | 4 (31) |  | 58 (58) | 8 (33) |  | 48 (59) | 18 (42) |  |
| **Current Smoker** | 20 (15) | 20 (18) | 0 (0) |  | 16 (16) | 4 (17) |  | 14 (17) | 6 (14) |  |
| **Former Smoker** | 34 (27) | 27 (24) | 7 (54) |  | 24 (24) | 10 (42) |  | 16 (20) | 18 (42) |  |
| **BMI, median [IQR] (kg/m2)*** | 30.9 [25.6-36.5] | 31.2 [25.3-36.7] | 29.6 [25.7-34.3] | 0.34 | 31.3 [26.2-36.7] | 29.3 [23.8-36.4] | 0.32 | 30.7 [26.6-36.7] | 31.1 [25.1-36.4] | 0.84 |
| **BMI cutoffs (kg/m2) (N (%))*** |  |  |  | 1.00 |  |  | 0.65 |  |  | 1.00 |
| **Non-Obese (<=30)** | 57 (47) | 51 (46) | 6 (46) |  | 45 (45) | 12 (50) |  | 37 (46) | 20 (47) |  |
| **Obese (>30)** | 65 (53) | 59 (53) | 6 (46) |  | 54 (54) | 11 (46) |  | 42 (52) | 23 (53) |  |
| **Comorbidities (N (%))*** |  |  |  |  |  |  |  |  |  |  |
| **HTN** | 75 (61) | 68 (61) | 7 (54) | 1.00 | 60 (60) | 15 (63) | 0.81 | 45 (56) | 30 (70) | 0.18 |
| **T2DM** | 50 (41) | 43 (39) | 7 (54) | 0.22 | 37 (37) | 13 (54) | 0.10 | 26 (32) | 24 (56) | **0.020** |
| **Asthma** | 22 (18) | 21 (19) | 1 (8) | 0.69 | 19 (19) | 3 (13) | 0.76 | 16 (20) | 6 (14) | 0.47 |
| **COPD** | 18 (15) | 15 (14) | 3 (23) | 0.38 | 11 (11) | 7 (29) | **0.043** | 11 (14) | 7 (16) | 0.79 |
| **Malignancy** | 16 (13) | 13 (12) | 3 (23) | 0.19 | 10 (10) | 6 (25) | 0.08 | 8 (10) | 8 (19) | 0.26 |
| **Cardiovascular Disease** | 18 (15) | 14 (13) | 4 (31) | 0.08 | 13 (13) | 5 (21) | 0.33 | 10 (12) | 8 (19) | 0.43 |
| **CKD** | 18 (15) | 14 (13) | 4 (31) | 0.08 | 11 (11) | 7 (29) | **0.043** | 8 (10) | 10 (23) | 0.06 |
| **HLD** | 39 (32) | 33 (30) | 6 (46) | 0.19 | 29 (29) | 10 (42) | 0.22 | 21 (26) | 18 (42) | 0.10 |
| **CXR by Total Score (N (%))** |  |  |  | **0.048** |  |  | **0.0005** |  |  | **0.004** |
| **0** | 0 (0) | 0 (0) | 0 (0) |  | 0 (0) | 0 (0) |  | 0 (0) | 0 (0) |  |
| **1** | 7 (6) | 7 (6) | 0 (0) |  | 7 (7) | 0 (0) |  | 7 (9) | 0 (0) |  |
| **2** | 26 (21) | 24 (22) | 2 (15) |  | 24 (24) | 2 (8) |  | 19 (23) | 7 (16) |  |
| **3** | 26 (21) | 25 (23) | 1 (8) |  | 24 (24) | 2 (8) |  | 20 (25) | 6 (14) |  |
| **4** | 28 (23) | 26 (23) | 2 (15) |  | 22 (22) | 6 (25) |  | 17 (21) | 11 (26) |  |
| **5** | 17 (14) | 12 (11) | 5 (38) |  | 11 (11) | 6 (25) |  | 8 (10) | 9 (21) |  |
| **6** | 20 (16) | 17 (15) | 3 (23) |  | 12 (12) | 8 (33) |  | 10 (12) | 10 (23) |  |
| **RT-PCR Results (N (%))*** |  |  |  | 0.75 |  |  | 1.00 |  |  | **0.022** |
| **Negative** | 37 (31) | 34 (31) | 3 (23) |  | 7 (29) | 30 (31) |  | 7 (17) | 30 (61) |  |
| **Positive** | 84 (69) | 74 (69) | 10 (77) |  | 17 (71) | 67 (69) |  | 35 (83) | 49 (39) |  |
| **CXR Sextus Score ≥ 3** | 91 (73) | 80 (72) | 11 (85) | 0.51 | 69 (69) | 22 (92) | **0.037** | 55 (68) | 36 (84) | 0.09 |
| **CXR Sextus Score ≥ 5** | 37 (30) | 29 (26) | 8 (62) | **0.020** | 23 (23) | 14 (58) | **0.002** | 18 (22) | 19 (44) | **0.014** |
| **Presenting CXR Sextus Score (0-6), median [IQR]** | 4.0 (2.0-5.0) | 3.0 (2.0-5.0) | 5.0 (4.0-5.0) | **0.048** | 3.0 (2.0-4.0) | 5.0 (4.0-6.0) | **0.0005** | 3.0 (2.0-4.0) | 4.0 (3.0-5.0) | **0.004** |
| **First CT Sextus Score (0-6), median [IQR]** | 4.0 (2.5-5.0) | 4.0 (2.0-5.0) | 5.0 (4.0-6.0) | **0.075** | 3.0 (2.0-5.0) | 5.0 (3.5-6.0) | **0.005** | 3.0 (2.0-5.0) | 5.0 (3.0-6.0) | **0.005** |

Categorical variables are expressed as counts and percentages. Continuous variables are expressed as medians with interquartile ranges [IQR]. Significant p-values (<0.05) are bolded; **AMA=against medical advice;** CXR=chest radiography; CT=computed tomography; BMI=body mass index; HTN=hypertension; T2DM=type II diabetes mellitus; COPD=chronic obstructive pulmonary disease; CKD=chronic kidney disease; HLD=hyperlipidemia

*4, 2, 1, 3 patients were missing smoking history, BMI, comorbidity, and RT-PCR data, respectively

**Supplemental Digital Content 2. Table which illustrates patient demographics, clinical findings, and presenting CXR scores in relation to outcomes of interest. docx**
